# How to Get a Grip on Processes of Communalization and Distinction in Group Interactions—An Analytical Framework

**DOI:** 10.3389/fpsyg.2022.786685

**Published:** 2022-02-22

**Authors:** Kristin Weiser-Zurmühlen

**Affiliations:** Department of German Studies, School of Humanities and Cultural Studies, University of Wuppertal, Wuppertal, Germany

**Keywords:** framework, Conversation Analysis, Positioning Theory, group interaction, qualitative research methods

## Abstract

This article proposes an analytical framework that combines Conversation Analysis, Positioning Theory, and Stance Analysis to study communalization and distinction as basic interactive mechanisms within group interactions. The framework is based on the premise that participants in multi-party interactions constantly manage the local demands of the ongoing conversation and turn-by-turn talk as well as implicitly or explicitly evoked references to global discourses, which in turn are closely related to the topic currently discussed. By considering both micro- and macro-contextual features in the analysis of group interactions, it is possible to reach a deeper understanding of dynamic group activities. The framework has been empirically developed based on data from a study on epistemic positioning practices in adolescents’ group interactions about popular TV series in Germany. The data comprises ten videotaped focus group discussions that have been elicited in a school context. By applying the framework to the analysis of a single case from the corpus, insights can be gained, both on how group members’ finely adjust their epistemic and evaluative stances as well as on how the participants themselves interactively link their stances to broader discourses.

## Introduction

In this paper I present my methodological framework for analyzing communalization and distinction in group discussions. When studying groups, it is essential to account for the characteristic features of multi-party interactions as opposed to dyadic interactions. When more than two people engage in talk, taking, maintaining, and relinquishing the floor for turns at talk becomes more complex than in dyadic conversation (see Stivers, 2021). Addressing several interlocutors with different knowledge, relationships as well as roles, rights, and obligations requires a speaker to employ diverse communicative actions and practices to participate in the ongoing interaction. In addition, participants might form and change alliances to achieve communicative actions. There are some suggestions for applying Conversation Analysis as a useful tool for studying group discussions on the micro-level of interaction, e.g., in order to analyze how group members organize turn taking activities (e.g., Gavora, 2015). Furthermore, Positioning Theory and its roots in discourse analysis is also used as a method to analyze multiparty interaction (e.g., Hirvonen, 2016). By approaching data with Positioning Theory, researchers seek to identify common orientations to what Bamberg (1997, p. 337) calls “level 3 positioning,” i.e., broader discourses shared by a social group (de Fina, 2013). But as Wetherell (1998) argues, these orientations are usually made relevant by the participants according to current situation and context. Thus, micro- and macro-scaled positionings are closely interlinked. However, little is known about how to analytically reveal how participants establish, change and negotiate intragroup similarities and differences by positioning practices on both the micro- as well as the macro-level of interaction. Conclusively, one method appears to be insufficient to describe the complexity of multi-party interactions.

As a solution, I propose a framework to capture this complexity by combining ethnomethodological Conversation Analysis (Sacks et al., 1974; Sidnell, 2013), Positioning Theory (Harré and van Langenhove, 1999; Harré et al., 2009), and Stance Analysis (Du Bois, 2007) and systematically relating the findings to each other. I have developed the framework empirically from a corpus of video recordings I took of adolescent focus groups in Germany discussing TV series. I instructed the group members to converse about whatever they chose, as long as it was related to the topic of *popular TV series*, e.g., which series they enjoyed watching, why or why not etc. Using Conversation Analysis, I analyzed how the participants orient to this instruction, managing topic-related participation in the ongoing interaction on the one hand while avoiding excluding other group members on the other hand. I used Positioning Theory and Stance Analysis to describe how they position themselves and others by taking epistemic and/or evaluative stances toward TV series. Considering these conditions, my videotaped multi-party interactions can be characterized as highly dynamic, driven by emerging and changing communalization and distinction processes within a (sub-)group. My framework helps to analytically describe these processes of how participants manage similarities and differences on various levels.

With the terms *communalization* and *distinction*, I refer to the verbal and non-verbal displays interlocutors use to signal the degree of how close, similar and/or agreeable they perceive their relationship to the other participants. It is an analytical notion to describe how group members manage similarities and differences on different layers (see also Weiser-Zurmühlen, 2021). On the one hand, this pairing refers to group dynamics for forming alliances alongside the distribution of knowledge as well as diverse assessments concerning series. On the other hand, this pairing is related to Bucholtz and Hall (2005, p. 599) differentiation between “adequation and distinction” within their framework for analyzing identity in interaction, in which it is part of several relational axes along which interlocutors might construct their identities.

Although I do not explicitly examine identity constructions in this paper, communalization and distinction cannot be separated from identity issues. Especially when interlocutors talk about their taste in aesthetic works like TV series, they tell each other which series they (do not) know and (dis)like, thereby continuously displaying a certain facet of their self. However, taste is not merely a question of individual preference but is also embedded in broader conceptions of “good” and “bad” taste shared by a community (see Bendix et al., 2012, p. 313). Individuals’ aesthetic preferences are usually constructed relationally to other people’s tastes. Interlocutors position themselves and others by comparing and adjusting to each other’s evaluations of media products, establishing their mutual orientations to normativity, and considering moral ascriptions of certain products and their consumers. These positions are implicitly related to politically relevant phenomena like the social distinction in the sense of Bourdieu (1984). The participants contribute to these underlying requirements on the micro-level of the interactional situation and the macro-level of societal discourses by establishing different degrees of communalization and distinction.

In this paper, I introduce my framework in the following way: In section “Methodological Approaches for Studying Dynamics in Group Interactions: Conversation Analysis, Positioning Theory and Stance Analysis,” I first discuss the concepts and their interconnectedness alongside how each analytic approach understands the three key concepts context, identity, and morality. I explain how to use these concepts for connecting communalization and distinction on different levels before I present and summarize the analytical framework in section “Proposal of a Framework: Positioning Practices for Establishing Communalization and Distinction.” I then apply the framework to a single case from the data set on the TV series *Game of Thrones* (section “Applying the Framework to a Single Case”), analyzing in detail communalization and distinction processes on the micro- and macro-level of interaction. With the analysis, I aim to demonstrate that linking global aspects of the currently discussed topic to the local level of interactive practices is important for understanding group formation dynamics.

## Methodological Approaches for Studying Dynamics in Group Interactions: Conversation Analysis, Positioning Theory and Stance Analysis

In this section, I introduce each methodological approach briefly before I discuss their contributions to analytical features of context, identity construction and morality, and normativity in group interactions.

Conversation Analysis has its roots in ethnomethodology. Its core view of interaction is that it is continuously brought into being by the interlocutors in their turn-by-turn talk. This proposition follows ethnomethodologists in assuming that social structures are not objectively (pre-)determined, but that interlocutors actively produce and reciprocally confirm them. Garfinkel (1967) refers to this process as an “ongoing accomplishment,” meaning that the members of a society construct their social reality by means of everyday and routinized practices. According to Garfinkel (1967, p. 118), these practices are “seen, but unnoticed” as they are mostly performed habitually and grounded in processes of social and cultural socialization. As a result, ethnomethodological researchers seek to reconstruct these practices by asking how interactants establish sense and order through them. Based on these principles, Harvey Sacks et al. (1974) developed Conversation Analysis to study the sequential structure of everyday conversations. Following the premise that “[there is] order at all points” (Sacks, 1984, p. 22), Sacks et al. (1974) demonstrate that interactants systematically manage turn taking-related rights and obligations. Its microanalytic focus is the most characteristic feature of ethnomethodological Conversation Analysis, i.e., to “look at conversations as if through a ‘microscope”’ (Heller, 2014, p. 224). Since then, Conversation Analysis has further developed and, in addition to analyses of local phenomena, has also been used to describe larger and more global structures such as communicative genres (Günthner and Knoblauch, 1997) or discourse units such as narrations (Hausendorf and Quasthoff, 2005), explanations (Morek, 2012), and arguments (Heller, 2012). I draw on the conversation analytic constructivist perspective view on interaction as well as its micro-analytical focus as key thoughts for the framework.

Positioning Theory was first introduced by Hollway (1984) with reference to Foucault (1972) notion of *subject position* as an analytical tool for capturing the interactive constitution of gender. Hollway (1984) posits that social discourses provide a selection of certain positions for men and for women: “Discourses make available positions for subjects to take up. These positions are in relation to other people. Like the subject and object of a sentence [.] women and men are placed in relation to each other through the meanings which a particular discourse makes available” (p. 236). She thus argues that while social discourse might pre-structure certain positions for individuals, they can actively choose or reject these positions in social encounters. Continuing Hollway’s argumentation, Davies and Harré (1990, p. 48) understand positioning as local references to social discourses, which they call “story lines”. Since then, the Positioning Theory has been systematized along different dimensions and forms of positioning (Harré and van Langenhove, 2007; Harré et al., 2009), e.g., positioning analysts distinguish between self- and other-positionings as well as their sequential placement. For the framework, I consider the ability of Positioning Theory to capture references to macro-societal structures as a fruitful approach.

Stance Analysis has been mainly shaped by Du Bois (2007). He draws on the Positioning Theory of Harré and colleagues and suggests focusing on stance as a small unit that interlocutors might use to establish positions. He defines stance as “a public act by a social actor, achieved dialogically through overt communicative means, of simultaneously evaluating objects, positioning subjects (self and others), and aligning with other subjects, with respect to any salient dimension of the sociocultural field” (Du Bois, 2007, p. 165). According to him, stance taking can be modeled as a triangular framework, working by the following mechanism: Subject 1 assesses a certain object whereby the individual positions themselves, followed by subject 2’s evaluative positioning, thereby aligning with the first person’s stance. The concept of stance taking has been adopted for several purposes (see e.g., Spitzmüller, 2013 on language ideologies; see also contributions in Englebretson (2007) and in Jaffe, 2009, as well as Chindamo et al., 2012). The stance triangle is embedded in the *theory of stance*, presuming that speakers align themselves to the linguistic units of other speakers (morphosyntax, lexis, and prosody). By syntactically paralleling the utterances, their paradigmatic relationship can be analytically explored, and researchers can work out nuanced meanings between the two forms. Du Bois calls this process “dialogic syntax” (Du Bois, 2007, p. 160). However, this part of the theory of stance will not be considered for this paper. Instead, I use Du Bois’ modeling of interlocutors positioning via evaluative stance taking toward an object of conversation appears to link Conversation Analysis and Positioning Theory in the framework.

### Analysis of Contextual Relations in Group Interactions: The Role of Micro- and Macro-Context

The three methods highlight analytical concepts for identifying the sequential order of communicative actions, such as the next-turn-proof-procedure (Sacks et al., 1974, p. 728),^1^ distinguishing between first, second, and third order positions^2^ (Harré and van Langenhove, 2007, p. 396) or between stance lead and stance follow^3^ (Du Bois, 2007, p. 165). Moreover, all approaches share the assumption that, to study interaction, the analyst needs to consider the interactive context in order to interpret an individual’s utterances. However, conversation analysts do not understand context as a kind of pre-determined “bucket” in which actions can be poured. They assume that individuals act context-sensitively and interpret preceding actions according to their everyday knowledge, ascribing it to be socially and culturally shared, thus carefully adjusting their subsequent utterances to precisely this context. From this perspective, context cannot be viewed as something predominantly given, but as a reflexive and social entity actively produced by the participants (Bergmann, 1994, p. 8). Interlocutors use contextualization cues (Cook-Gumperz, 1978; Gumperz, 1992) and draw on different communicative resources (e.g., prosody, lexical choices, or gestures) to signal to each other their current understanding of the interactional situation (see also Du Bois, 2007, p. 146 for the status of contextualization cues in his framework). Speakers reciprocally orient toward each other and design their utterances for other participants to understand them. Hence, when analyzing social interaction from an ethnomethodological perspective, researchers use the same analytic means to which speakers themselves have access, namely closely observing and interpreting co-participants’ actions.

In contrast to Conversation Analysis, especially early positioning analysts viewed contextual structures as to some degree pre-determined (see also Deppermann, 2015, pp. 370–372), grounded in Foucault’s structuralist notion of *subject position* which regards discourses as accountable for distributions of knowledge and power. Gee (1996) called these discourses D-discourses, defining them as “a socially accepted association among ways of using language, other symbolic expressions, and artifacts [.] that can be used to identify oneself as a member of a socially meaningful group or ‘social network”’ (Gee, 1996, p. 131). Davies and Harré elaborated on this idea of positioning as interactional references to broader discourses. They illustrated it by using examples of constructed dialogues between fictional characters representing social categories (“Sano” and “Enfermada,”, see Davies and Harré, 1990, pp. 55–58) as well as possibilities of gender-related readings of a fictional narrative (see Davies and Harré, 1990, pp. 60–61). However, other researchers—especially researchers working with Conversation Analysis—reject such an essentialist view and assume discursive positions to be a matter of the participants’ construction and interpretation (Wortham, 2000; Lucius-Hoene and Deppermann, 2004; Bamberg and Georgakopoulou, 2008).

Conversation analytic research on group interaction has also shed light on micro-scaled features affecting the group’s interactional process. For example, Gibson (2003) introduced the concept of *participation shift*, which encompasses the turn-by-turn transformation of the participation framework in multi-party interactions. Gavora (2015) identified two interaction patterns—*Catalogue* and *Domino^4^* —in moderated focus groups, and Gatica-Perez et al. (2012) studied dynamic processes in small groups from a multimodal perspective, taking into consideration contextual features like conversational attention, turn taking and conversational floor as well as practices for addressing or interrupting other participants. Further research for describing these complex relationships has been conducted by Onwuegbuzie et al. (2009) who suggested the so-called “micro-interlocutor analysis” (p. 7) as a qualitative framework, using Conversation Analysis as a key method. Their framework allows collecting information about the participants’ order of responding to questions, the response characteristics as well as non-verbal communication aspects.

In my framework, I suggest that analysts can use contextualization cues, such as e.g., the responses to questions as suggested by Onwuegbuzie et al. (2009), in order to identify processes of micro-level communalization and distinction processes through closely examining the turn-by-turn-talk with regard to turn allocation, repair as well as communicative actions and practices. However, researchers who aim to identify interlocutors’ references to broader social discourses by using only Conversation Analysis face methodological limitations. For instance, some conversation analysts demand that certain social constructs such as gender ought to be considered only if analysts can show that participants display a local orientation to the existence of these social constructs (Schegloff, 1997, p. 180).^5^ This appears to be a methodological limitation, as participants can also orient to categories or attributes that are merely “invoked” (Wilkinson and Kitzinger, 2003, p. 174).

### Analysis of Identity Construction in Group Interactions

As speakers orient toward each other, they design their utterances based on their assumptions of who the co-interactants are and what they know to achieve mutual understanding and intersubjectivity (see also Du Bois, 2007, p. 140). Conversation analysts describe this process as *recipient design* (Schegloff et al., 1977). With recipient design, interlocutors show how they understand the other participants as well as how they themselves seek to be understood—or in terms of positioning, how they position themselves and others (Harré and van Langenhove, 2007, p. 398), thus constructing social identities.

Concerning the issue of identity construction, another methodological difference between Conversation Analysis and Positioning Theory can be pinpointed. Some positioning theorists such as Harré and van Langenhove (2007, pp. 399–404) assume that positioning can be performed intentionally or strategically. This idea appears to be hardly compatible with the antimentalist perspective taken by Conversation Analysis. Instead, from an ethnomethodological point of view, identity is regarded as a social construction (Berger and Luckmann, 1969; Antaki and Widdicombe, 1998) based on individual as well as mutual engagement in ascriptions and affiliations (Berger, 2010). For instance, Antaki et al. (1996, p. 489) show how a person’s identity may take a variety of different forms depending on the conversational context in which it is invoked. However, further developments of Positioning Theory claim that “a positioning view on self and identity is [neither] opposed to a static and essentialist view of identity [nor] does [it] locate identities in some abstract, integrated structure ‘behind’ discursive practice, but in what people observably do” (Deppermann, 2015, p. 370).

Linguistic researchers like Bamberg (1997), in their adoption of the positioning concept for analyzing narrative identities, distinguish between three levels of positioning: level 1 includes positionings of individuals within the narrated world; level 2 positionings are located on the interactional surface between interlocutors while level 3 positionings refer to “master narratives,” comparable to D-discourses (Bamberg, 2004, p. 225; see also: Bamberg, 1997; Lucius-Hoene and Deppermann, 2004; Bamberg and Georgakopoulou, 2008; de Fina, 2013). Another way to analyze identity displays is Membership Categorization Analysis (Schegloff, 2007). Applying this method, analysts can describe how participants position themselves and others in relation to more or less conventionalized social categories by invoking, emphasizing or rejecting certain category-related attributes (Wilkinson and Kitzinger, 2003; Stokoe, 2005; Liebscher and Dailey-O’Cain, 2007; Tirado and Galvez, 2008; Deppermann, 2013).

Another methodological discussion concerns the question of which kind of knowledge researchers can include for interpreting identity displays. Conversation analysts argue that researchers should draw almost exclusively on ethnographic knowledge in the sense of long-term observation of participants, thus gaining familiarity with the field (see Bamberg and Georgakopoulou, 2008, p. 379, Georgakopoulou, 2013, p. 106; Deppermann, 2013, p. 106). However, Bamberg and Georgakopoulou (2008) show that further knowledge concerning societal discourses is also necessary to interpret identity-constructive utterances in group interaction. In one of their group interviews, a male participant jokingly starts to sing the song *It wasn’t me* by Shaggy. The authors draw the analytical conclusion from “the meanings [.] this borrowing [from Shaggy] indexically evoke” that “both [the participant and Shaggy] engaged in women in largely hegemonic male ways and in (contradictory) denial of this engagement” (Bamberg and Georgakopoulou, 2008, p. 391). Hence, the authors consider the sequential context as well as the entire song text as a resource for their interpretation (see Bamberg and Georgakopoulou, 2008, p. 394, footnote 5). Since analysts are members of the same social community as the group participants, they are able to draw on shared knowledge (see also Garfinkel and Sacks, 1970); in this case about Shaggy as a public character as well as his performative display as a musician and as a heterosexual man.

For my framework, I follow an ethnomethodological perspective and do not consider identity-positionings as intentional or strategic. I assume that researchers can analytically reconstruct how individuals might use social categories in order to establish communalization or distinction with other participants, establishing ideas of sameness and distance e.g., by labeling social types or categories as *others* they distance themselves from Liebscher and Dailey-O‘Cain (2014); Günthner and Zhu (2016). Furthermore, I suggest that analyses need to systematically include socially shared knowledge amongst members outside of the interaction about the media product and the social and popular cultural discourse in which it is embedded.

### Analysis of Morality and Normativity in Group Interactions

There is a large body of research on the construction of norms and morality in interaction. Interlocutors might take what Rakoczy and Schmidt (2013) call a *normative stance*., i.e., they constantly balance what they themselves and other interlocutors can, should, must or may (not) do or say. From an ethnomethodological perspective, participants produce, confirm, and negotiate these stances that refer to morality and normativity discursively and contextually in interaction (see Bergmann, 2013; Günthner, 2013). However, analysts typically do not have access to normative orientations, as interactants rarely explicate them. Instead, interactants use strategies of indirect moralization. They merely allude to shared moral knowledge without making this explicit or morally load certain utterances (Bergmann, 2013, p. 45).

When interlocutors talk about media, such as TV series, they display their taste by comparing what they know and (dis)like about it. Both negotiating assessments and knowledge can have moral dimensions (Raymond and Heritage, 2006; Stivers et al., 2011; Sidnell, 2012) which group members might interactively address by treating something as (not) normal. To reconstruct normative references, researchers analyze the evaluative stances participants take toward an object of talk. However, since the stance triangle as suggested by Du Bois (2007) only encompasses the analysis of assessments (see section “Methodological Approaches for Studying Dynamics in Group Interactions: Conversation Analysis, Positioning Theory and Stance Analysis”), it has been further differentiated by means of analyzing claims, ascriptions, and rejections concerning a stance’s epistemic dimension (see Heritage and Raymond, 2005; Stivers et al., 2011; Kiesling, 2016).^6^ For example, from a conversation analytical perspective, Heritage (2012, p. 4) stated that interlocutors position themselves via epistemic stance-taking. By aligning their epistemic stances, participants locate themselves on an epistemic gradient of being more or less knowledgeable (K+ or K-) than their co-interlocutors concerning a certain territory of knowledge (Kamio, 1997). Assessments and epistemics are closely intertwined: How interlocutors distribute epistemic rights and obligations to assess something depends on the degree of displayed and/or assigned knowledge and strength of the evaluation (Raymond and Heritage, 2006), thus interactively orienting to epistemic authority (Mondada, 2013).

There is some research on references to moral discourses in group interactions. For instance, Hirvonen (2016) shows how groups establish and negotiate both institutional and conversational moral orders. Smithson (2000) argues that by applying Positioning Theory to focus group data, interactive features such as individuals dominating within a group can be related to tendencies toward normative discourses as well as managing conflicts and arguments. Similarly, Halkier (2010) analyses Danish women’s cooking practices and demonstrates how an analytical focus on self- and other positioning as well as on establishing alliances within a group can contribute to an understanding of group dynamics in interaction in terms of the negotiation of norms and their moral implications. Grønkjær et al. (2011) discuss how the degree of a group’s heterogeneity or homogeneity can influence the interactive construction of normality.

I integrate the analysis of the participants’ epistemic and evaluative stances in my framework, identifying how the participants display, negotiate, or reject their mutual understanding of socially and/or culturally shared (moral) assumptions and what they treat as normal or deviant. I argue that they display communalization and distinction with regard to how they position themselves and others to these morally loaded discourses.

## Proposal of a Framework: Positioning Practices for Establishing Communalization and Distinction

My framework is based on the proposition that participants in multi-party interactions constantly have to manage the local demands of the ongoing conversation and the turn-by-turn talk as well as the implicitly or explicitly evoked references to global discourses, which in turn are closely related to the topic currently under discussion. As group members might position themselves and others with respect to this topic through epistemic and/or evaluative stance-taking activities, I use Stance Analysis identify a stance’s target, which I call the *positioning object*.

For a detailed analytical access to the relationship between epistemic and evaluative positionings in terms of a specific positioning object, I suggest analyzing how participants, turn by turn, position themselves and others as more or less knowledgeable (*K*+ and *K –* in Heritage’s terminology) and communicate a more positive or rather negative assessment of a series. Depending on the interlocutors’ positioning along these two dimensions of epistemic and evaluative stance as, e.g., more knowledgeable and critical or less knowledgeable yet appreciative toward a series they use different practices for claiming, assigning, and denying epistemic knowledge and authority. Interlocutors functionalize these practices for establishing finely granulated communalization and distinction activities according to how the other participants position themselves and others, thus constructing identity facets (see section “Analysis of Identity Construction in Group Interactions”). These practices and actions in turn can be analyzed on the micro-level of interaction (see section “Analysis of Contextual Relations in Group Interactions: The Role of Micro- and Macro-Context”) by applying the sequential and context-sensitive methodology of Conversation Analysis.

However, the degree of specificity of the positioning object has consequences for the interlocutor’s stance-taking activities. Participants may not only position themselves with relation to a specific topic (e.g., a specific TV series like *Game of Thrones*), but may also establish an abstract positioning object which can be morally loaded or embedded within normative discourses (see section “Analysis of Morality and Normativity in Group Interactions”), e.g., concerning the depiction of series characters. This changes the scope of positioning: not only does specific knowledge or specific assessments of a series a play role, but participants may also bring higher-level knowledge elements and discourses into the interaction for negotiation. As most conversation analysts do not systematically include topic-related features in the analysis, I suggest that Positioning Theory enables researchers to take into account references to D-discourses.

**FIGURE 1:**
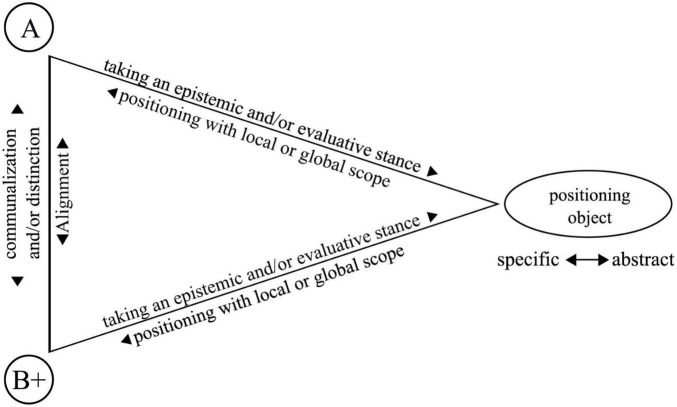
Proposal of an analytical framework (translated and retrieved from Weiser-Zurmühlen, 2021, p. 288).

My proposed framework for studying group processes and dynamics of communalization and distinction can be summarized as the following (see Figure 1): I assume a triadic relationship between interlocutors and their discussed thematic issues, similar to the stance triangle by Du Bois (2007). However, unlike Du Bois, my model also includes two or more participants, with one person displaying a primary position and other participants aligning themselves with this position. I assume that the participants initiate a positioning object by expressing their evaluative and/or epistemic stance toward the object implicitly or explicitly, by assigning it to others or asking others about it. In this way, they position themselves with local scope, i.e., in the context of the ongoing interaction situation. Applying Positioning Theory, researchers can also identify positions with global scope, i.e., related to discourses concerning the topic. I argue that researchers can integrate and reflect upon socially shared knowledge about these discourses in the analysis.

For practical analytical purposes, I suggest two steps: First, a micro-level analysis of communalization and distinction, and then a macro-level analysis. I analyze communalization and distinction on the micro-level by studying how alliances within the group are established and processed. Therefore, using Conversation Analysis, I examine and describe the group interaction’s sequential structure to identify communicative actions that participants deal with (e.g., finding consensus, disagreeing, remembering). Via Stance Analysis, I analyze the epistemic and evaluative stances the participants take toward the positioning object and set the stances in relation to contextual features. Thus, I can reconstruct which positioning practices the participants use depending on their epistemic status and (d)evaluation of a positioning object as well as how they display and negotiate identity facets via self- and other-positionings.

Second, I detect communalization and distinction on the macro-level by applying Positioning Theory and interpreting how the participants position themselves and/or others in (dis-)alignment with discourses and narratives organized around the positioning object. I study how they refer to underlying norms and moral understandings by addressing the positioning object. In the following, I will apply the framework to a single case of my data as an example.

## Applying the Framework to a Single Case

My data comprise a corpus of ten video recordings I took of adolescent focus groups in Germany discussing TV series. Most recordings took place in rooms provided by the students’ schools, such as empty classrooms or faculty rooms. The groups ranged from three to seven participants. All students voluntarily participated in the study and were required to give written consent for data collection, storage, and sharing. After instructing them to converse about the topic, I left the room and the participants organized their discussion by themselves, with the explicit request that they discuss the topic as a whole group and avoid schisming.

**FIGURE 2:**
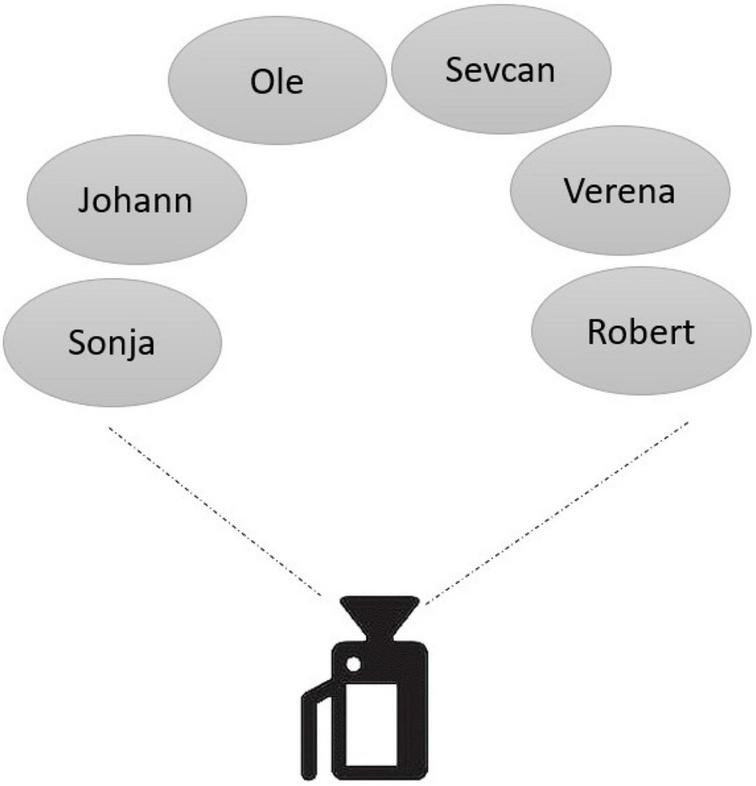
Arrangement of the group participants and the camera.

In the data extract selected for the analysis (Extract 1), the participants were in grade 11 at a German *Gymnasium* (secondary school). The group consisted of six members aged 17–18 years, of which three were male (Johann, Ole, and Robert) and three were female (Sonja, Sevcan, and Verena). The participants were arranged in a semi-circle in front of the camera (see Figure 2). Their teacher allowed the students approximately 60 min to participate in the study, as the study took place during a German lesson. The extract chosen from the group discussion deals with the series *Game of Thrones*, which proved to be a controversial series in the group as there was fierce disagreement regarding its quality.

**EXTRACT 1:**
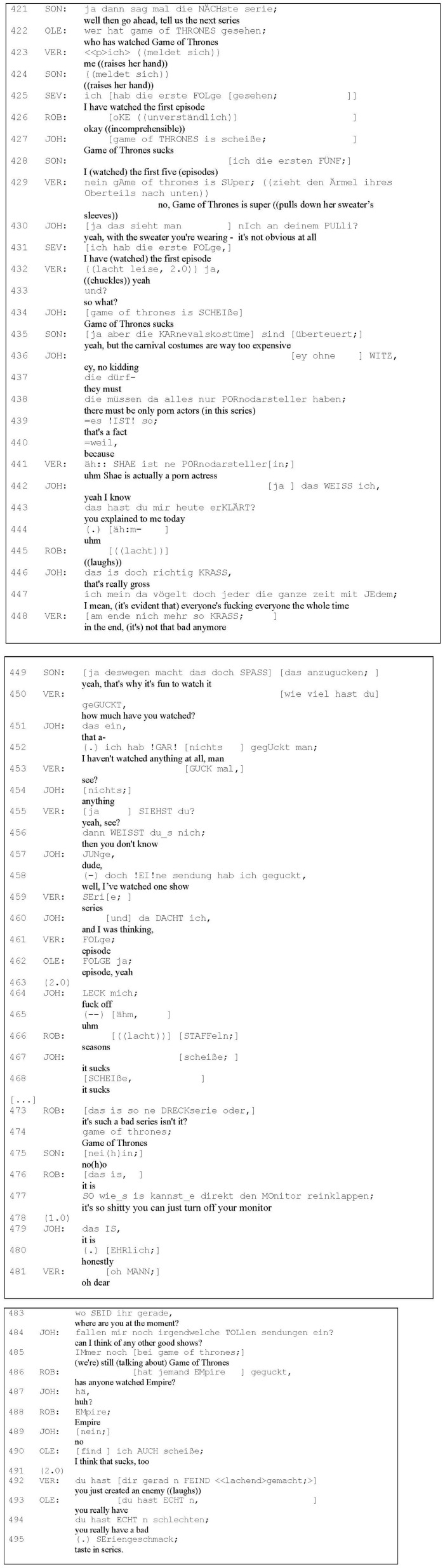
Group OST11, 25‘46“–26‘:39“. Reproduced with permission from Kristin Weiser-Zurmühlen, available at https://www.degruyter.com/document/isbn/9783110727845/html.

The following transcript includes enumerated utterances from all of the group participants Sonja (SON), Ole (OLE), Verena (VER), Sevcan (SEV), Johann (JOH), and Robert (ROB). For a visual impression of the participants, see Figure 2. The extract was transcribed according to the conventions of *Gesprächsanalytisches Transkriptionssystem 2* (GAT 2; see Selting et al., 2011). This method captures not merely the verbal content but also non-verbal and prosodic features and their alignment with the verbal utterances. The German transcription conventions include capital letters to index emphasis of syllables, punctuation to show rising or falling intonation at a turn’s end and square brackets to visualize overlaps and parallel speaking. With the English translation below each line, I try to capture the content and tone of the utterances but do not copy prosodical features.

The sequence starts with Ole asking who has watched the series *Game of Thrones*, followed by the others responding with different evaluations of the series (lines 422–434). Johann justifies his negative evaluation by referring to the frequent depiction of sex in the series; the depiction of sex then becomes the object of interactive negotiation (lines 435–449). As Johann announces that he has not watched a single episode of the series (lines 450–454), Verena challenges his assessment with regard to his epistemic status (lines 453, 454), followed by a repair sequence on the correct term to be used for an episode (lines 455–466). While Johann and Robert continue assessing the series negatively, Ole and Verena discuss the series in terms of content (lines 469–472; this part is left out of the transcript with considerations of space). The sequence ends with the group searching for a new topic to talk about (*Empire*) (lines 482–486), leading to a mocking sequence about Johann’s general taste in series (lines 487–495).

I organize the analysis alongside the steps as suggested in section “Proposal of a Framework: Positioning Practices for Establishing Communalization and Distinction”: First, I focus on the communalization and distinction on the micro-level, analyzing the sequential organization of turns, stance taking activities and positioning practices (section “Communalization and Distinction With Local Scope: Forming Interactive Alliances”). Second, I describe communalization and distinction on the macro-level by analyzing how the participants position themselves and others to discourses and narratives around series (section “Communalization and Distinction With Global Scope: Referring to Broader Discourses”). With the analysis, I aim to demonstrate two issues: On the one hand, I show how the participants establish communalization and distinction by forming two alliances. I reconstruct how they use a positioning practice I refer to as *challenging the epistemic authority for evaluation*. On the other hand, I show how communalization and distinction are also driven by identity work, morality, shared norms, and narratives around the conversational topic of the series.

### Communalization and Distinction With Local Scope: Forming Interactive Alliances

At first sight, there are two subgroups who display different opinions about the series’ quality, with one group in agreement about its brilliance (Ole, Verena, and Sonja) and the other about its dissatisfactory quality (Johann and Robin). In this extract, Sevcan explains to have watched the first episode, but she does not share her evaluation. Applying now a conversation analytical perspective on the extract, I describe how the participants design their utterances and produce a shared understanding of the current situation. I show how they mutually shift from displaying epistemic stances to evaluative stances which contributes to the emergence intergroup distinction.

Taking a look at the start of the sequence, Ole suggests talking about *Game of Thrones.* Although he designs his turn as an open question about the other group members’ reception of the series, the participants start managing dissensus about their appreciation of the series during the course of the sequence. However, Ole’s turn construction in terms of recipient design reveals that he assigns the other participants general knowledge about the existence of the series, probably due to its popularity. Other initial sequences in my data corpus usually start with formulations like “do you know series X?”; Ole, however, formulates the question as “have you watched series X?”. Besides merely answering the question, e.g., by raising his hand (like Sonja and Verena), or by stating how many episodes he has watched (like Sevcan and Sonja) (lines 425, 428), Johann treats Ole’s first turn as an invitation for evaluation by producing a negative assessment: “Game of Thrones sucks” (line 427). This negative assessment provokes a contradiction by Verena (line 429). Despite his utterance overlapping with Sonja and Sevcan sharing how many episodes they have watched, the other participants pick up Johann’s turn for further reaction. Thus, the group renews the context and shifts from (potential) exchange about the series’ content to rating its quality. They open the interactional floor for managing communalization and distinction through the (dis)agreeing assessments. Consequently, the participants treat *Game of Thrones* as a positioning object, i.e., an object toward which one can take an epistemic and evaluative stance, thereby positioning oneself and others.

**FIGURE 3:**
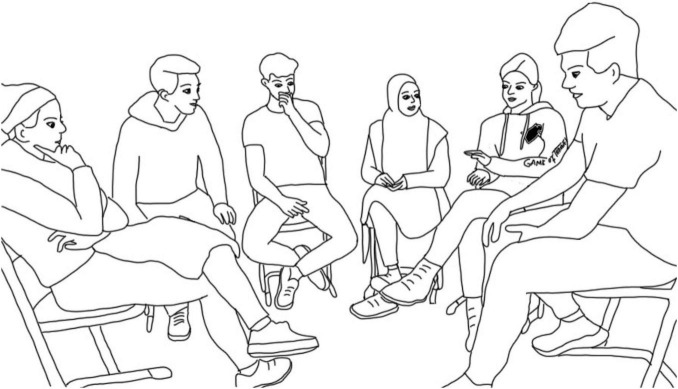
Verena pulls down the sleeves from her sweater, revealing it as part of the *Game of Thrones* merchandise.

I analyzed how the group’s participants throughout my data organize their stances in a typical way around the positioning object, using recurrent positioning practices. Verena uses a positioning practice I call *challenging one’s epistemic authority for evaluation*, which unfolds as the following interactive pattern: After a group member evaluates a series negatively and provides their utterance with high epistemic certainty, yet apparently not grounded in sufficient epistemic status, other group members might deny the participant their epistemic authority for assessment. In what follows, I reconstruct the sequential unfolding of the positioning practice and describe how it is related to contextual features and identity work:

Initially, Johann rates the series in a very negative way (line 427), whereupon Verena seeks eye contact with him, contrasting his statement with an explicitly positive evaluation (line 429). Almost at the same time, she pulls down the sleeve of her sweater (line 429). Johann also refers to the garment by ironically commenting on her evaluation: “yeah, with the sweater you’re wearing—it’s not obvious at all” (line 430). Using the video as an interpretational resource (see Figure 3), Verena’s sweater is apparently part of the *Game of Thrones* merchandise. This is where I suggest applying a conversation analytical view of context (section “Analysis of Contextual Relations in Group Interactions: The Role of Micro- and Macro-Context”) since Verena’s clothes as an object of joint attention are actively brought in the interaction and reflexively confirmed as the current context, shaping the next context. Against the background of identity work and social categorizations (see section “Analysis of Identity Construction in Group Interactions”), the sequence can be understood as follows: Johann positions Verena (and she positions herself) implicitly as a “fan” of the series, which makes her positive assessment of the series expectable. Additionally, she—as an assigned member of the social category *fan*—claims the right to criticize and doubt his expertise for judging the series, positioning him in turn as less knowledgeable (*K*-). However, the interpretation that people wearing merchandise are likely to be a fan of this series and displaying this identity facet for others on their bodies is not only grounded in the groups’ treatment of the garment but also in the researcher’s background knowledge about media industries (see section “Analysis of Identity Construction in Group Interactions”).

Johann argues against this positioning several times during the course of the sequence by justifying his negative assessment regarding the “fact” that all actors in the series were “porn actors” (line 438). He underlines his statement with a very strong emphasis on the verb “IS,” signaling a high epistemic certainty (line 439). Verena interrupts his justification (line 440), ignoring his negative evaluation and instead of agreeing with him by confirming that the actress portraying Shae^7^ was a pornographic actress (line 441). Here, it can be concluded that Verena attempts to establish a small degree of communalization with him by acknowledging the content of his utterance, but not the underlying evaluation. But Johann does not ratify this attempt, since he rejects the K- status implicitly attributed to him with reference to a former joint conversation (lines 442–444). Instead, he keeps on demonstrating to possess the epistemic authority to judge the actors’ activities and the apparent focus on sex in the series (lines 446–447) via his evaluative stance, thus maintaining the distinction between Verena and himself.

Finally, in two steps, Verena challenges Johann’s claimed epistemic authority for assessing the series’ quality. First, she asks him how much of the series he has watched (line 450). As he has apparently not watched a single episode (lines 452, 454), thus violating the preference for displaying an evaluation grounded in sufficient epistemic access (see Enfield, 2011, p. 202), she challenges him using the formulation: “yeah see? you don’t know;” (lines 455–456). Johann in turn modifies his epistemic status to the extent that he has watched one “broadcast” (line 458), but he does not change his judgement^8^ (lines 467, 468). Verena, followed by Ole and Robin, initiates repair of his lexical choice (lines 461, 462, 466). However, there is no repair uptake [following the preference structure for self- over other-initiated repair, see Schegloff et al. (1977), p. 374]: Johann continues to use the now-corrected term “broadcast”.^9^ His answer (“fuck off”, line 464) is not designed in a face-saving way—albeit framed jokingly. He does not downgrade his evaluation after Verena’s challenge, but merely modifies his epistemic stance (lines 452, 458). Some other group members attempt to reframe Johann’s negative assessment as well as to build him “bridges” for downgrading his extreme evaluation. For example, Verena and Sonja ratify his assertion that the series includes pornographic elements (rather than contradicting him). However, they interactively deal with it differently: While Verena declares it to be no longer applicable to current episodes (line 448), Sonja reinterprets Johann’s negative reason for evaluation positively, strengthening that she enjoys the display of sexuality in the series (line 449).

Both subgroups achieve intragroup communalization by agreeing and strengthening each other’s positionings while at the same time maintaining intergroup distinction for instance as Robin fosters Johann’s evaluation: “it’s such a bad series, isn’t it?” (line 473) while Sonja contradicts fiercely (line 475) which then finally culminates in jokingly questioning Johann’s general taste (lines 493–495). The distinction cannot be easily resolved due to different epistemic positionings: The participants who like the series ground their expertise in displaying their knowledgeability of the *Game of Thrones* universe. In this extract, this can be seen by the group members implicitly referring to background knowledge about the actors (line 441) and merchandise (line 435). In contrast, Johann and Robin who position themselves as critics by drawing on second-hand information admit to having watched only one or a few episodes.

### Communalization and Distinction With Global Scope: Referring to Broader Discourses

Johann grounds his negative evaluation of the series’ overt displays of sexuality in morality, i.e., he frames it as something inappropriate for the media production. This is where my framework allows for including broader discourses in the analysis (see section “Analysis of Contextual Relations in Group Interactions: The Role of Micro- and Macro-Context”): Aesthetic products can be regarded as embedded in normative discourses, as Bourdieu (1984) studies about social distinction and class demonstrate. Series, like other media products, can be divided into “high” and “popular culture,” with aesthetic artifacts associated with high culture commonly being socially deemed more appreciable and acceptable as leisure activities (see e.g., Buhmann et al., 2015, p. 7). In turn, consuming products identified as “popular” coincides with moral implications on both the products and the recipients (Fiske, 2011, p. 102). However, several academic fields, such as Cultural Studies, emphasize the recipients’ active and productive participation in popular culture by using it for their own identity work and appropriation (Jenkins, 1992; Johnson, 2006). Furthermore, the view on formats traditionally devalued as “popular” changes: As for TV series, for instance, certain kinds of shows have been categorized as Quality TV (see Thompson, 1996; Blanchet, 2011), thus attempting to upgrade the image of television shows.^10^ However, Cultural Studies understand popular culture as an opportunity for recipients to deal with media affordances (Gibson, 1979) independently and creatively. Starting with Stuart Hall (1980) encoding-decoding model, it has become commonly accepted that encoded media meanings do not necessarily have to be adopted by users for them to negotiate these meanings in context. Researchers stress that media recipients choose which features to treat as relevant for their identities (see Hepp et al., 2015).

Including these considerations in the analysis, Johann’s moral positioning and his refusal to relativize it, even after having been challenged on the surface (he has not sufficient epistemic access), become explicable: He distances himself not only from the other group members and their taste in this series, but also from a certain associated D-discourse (section “Analysis of Contextual Relations in Group Interactions: The Role of Micro- and Macro-Context”): displays of sexuality can be included in the perception of something being popular, mainstream, and not of high (cultural) value. Thus, he also positions himself as not *that* kind of series recipient in the sense of a “category-based denial” (Stokoe, 2010, p. 69), which can be seen in his distancing himself from Verena as a fan. Yet, morality is actively brought into being by the participants (see section “Analysis of Morality and Normativity in Group Interactions”), negotiated interactively and even appropriated against hegemonical readings, as Sonja uses the depiction of pornography—as something not ascribed to high culture—as a means of entertainment and as a reason for watching the series. To interpret this positioning, researchers need to explicitly include knowledge that is not grounded in the ethnography of the group interaction, but in socially shared knowledge about media discourses and norms (see also the “Shaggy”-example in section “Analysis of Identity Construction in Group Interactions”).

In sum, the participants establish the intergroup distinction and communalization among the subgroups’ members at the beginning of the sequence and they constantly uphold and reinforce it. However, not only do the different epistemic positionings contribute to their maintenance but two different layers of morality addressed by the interlocutors also seem to interact here: On the one hand, Johann does not follow the moral duty to ground his epistemic authority in sufficient epistemic access, which is interactively sanctioned by challenging his positioning. On the other hand, Johann grounds his positioning in moral assumptions about pornography as inappropriate for a TV series. He treats this assumption as shared with the other participants, but is contradicted once again by Sonja who enjoys the series’ display of pornography.

## Discussion

I propose to include communization and distinction as relevant phenomena in the analysis of group interactions, as they are phenomena to which participants regularly orient. My framework helps to understand how interlocutors negotiate intragroup similarities and differences at different levels. As my analysis demonstrates, communalization and distinction can be located at the micro-level of interactional organization as well as at the macro-level of societal and ideological discourses. Both the micro- and macro contexts are intertwined. For instance, on the one hand, interlocutors use positionings to accomplish communicative actions, namely dealing with dissent and face work; on the other hand, the students also position themselves beyond the interaction situation and implicitly refer to media-related norms and ideologies. Both forms of positionings relate to issues of identity and morality:

Johann constructs his identity in terms of expertise, as someone with the epistemic authority to judge the quality of the series. Through the positioning practice of *challenging one’s epistemic authority*, Verena questions not only his right to assess but also his identity construction as an expert, positioning him as less competent than he claims to be. In contrast, he implicitly positions Verena as a fan of the series and, at the same time, as distanced from the social category “fan.” Moreover, Johann positions himself as someone with a “good” taste who rejects inappropriately designed and narrated media products and their reception—and thus also implicitly their recipients. Researchers can study this identity-related distinction between the group members by tracing the sequential course of the interaction as well as considering socially shared knowledge about connotations of “being a fan” and ideological media-related discourses.

By relating micro- and macro-contexts, morality appears to be essential for positioning dynamics in group interactions. However, the participants make morality relevant on different levels. Displaying epistemic authority to assess something without sufficient access is a moral problem at the level of interaction (see Heritage and Raymond, 2005; Enfield, 2011), as assessments tend to be no longer ratified by other participants when they are obviously not grounded in sufficient epistemic access. Thereby, Verena positions Johann implicitly morally inferior because he violated interactional moral orders. On the other hand, Johann positions himself as morally superior by assessing the series and its apparent pornographic content, grounding his position in ideological discourses. The analytical finding that the participants make morality relevant on different levels helps to understand why both intergroup distinction as well as intragroup communalization are upheld and fostered during the course of the interaction: The subgroups apply different standards and justifications for their expressions and moral judgments and accordingly construct different identities, which in turn are interactively addressed and treated differently.

In this case study it becomes clear that in multi-party interactions participants face complex positioning possibilities. The more—diverging—positionings there are, the more the interlocutors have to take into account with whom they commune or from whom they distinguish themselves when they take an evaluative stance toward the topic. Their positioning can be grounded in communicative actions such as face work or managing humorous dissent, but it can also refer to broader discourses. In this way, participants have a complex range of possibilities to form, consolidate, or change alliances with other group members. They can orient themselves in a finely granulated way to a large selection of positions and can thus position themselves or others.

This complexity can be grasped analytically by examining group data with the presented three methods and then relating the results to each other. In the following, I discuss which method is suitable for considering which aspect and how to deal with possible incompatibilities between them. Combining Conversation Analysis and Positioning Theory could lead to what Deppermann (2015, p. 381) calls the “micro-macro problem” (see also Habscheid, 2000; Day and Kjaerbeck, 2013). Conversation Analysis focuses on predominantly local practices on the micro-level of the interactional situation. Epistemic positionings such as the one between Verena and Johann, grounded in the sequential course of the conversation and functionalized to distinguish between the two, can thus be reconstructed in detail. References to ideologies in the sense of D-discourses, on the other hand, are only to be included if the participants orient to them (see section “Analysis of Contextual Relations in Group Interactions: The Role of Micro- and Macro-Context”). Hence, Positioning Theory offers a fruitful view of individuals’ orientation to higher-level structures. Additionally, although some conversation analysts take topics into account, there is little work on the sequential unfolding of topics (cf., Maynard and Zimmerman, 1984), including narratives and discourses. This sequential analysis of interlocutors highlighting certain aspects of topics in interaction is precisely what Stance Analysis offers: studying the relationship between participants and a somehow shaped “topic of conversation” interlocutors orient to as conceptualized in Du Bois’ stance triangle.

I include both the concepts of stances and positionings into my framework because they offer a finer distinction in terms of their reference point. Positionings refer to more abstract entities than social discourses while stances refer to relationships between “speakers and speakers and conversations” (Kiesling, 2011, p. 2). In contrast, merely analyzing positionings and stances does not follow the constructivist view on context as Conversation Analysis does. Du Bois in particular only considers a few previous and next turns for his analyses and focuses mainly on syntactical alignment. Indeed, some researchers argue that Stance Theory and Conversation Analysis cannot be combined. However, Haddington shows how to understand stance taking as an “activity” in a conversation analytic sense. When stance can be understood as “the speakers’ subjective attitudes toward something,” stance *taking* “can be understood as a dialogical and intersubjective activity” (Haddington, 2004, p. 101). Moreover, Conversation Analysis helps to describe positionings and how they are conveyed multimodally. The rich conversation analytic research on interactional phenomena, such as recipient design, repair, etc., provides useful descriptive tools for the variable interactive resources conversational partners use to shape their stances and positionings. For example, prosody in the form of very strong stresses plays a central role in Johann’s epistemic positioning while Verena’s clothes become object of negotiating positions.

That identity and morality play such a central role is a finding I inductively grounded using Conversation Analysis as an approach to the data. I have reconstructed sequentially and systematically which orientations and topics are relevant for the participants. Thus, I described how they achieve communalization and distinction in a nuanced way by referring to different contexts and shared knowledge about discourses, identity facets and moral implications. While not all topics are as morally loaded as the topic presented here, it can still be assumed that these three aspects are important in other group contexts from the participants’ perspective. Especially since both identity and morality are rarely foregrounded interactionally and explicated communicatively (see Antaki et al., 1996 on identity; Bergmann, 2013 on morality).

With my approach, I aim to shed light on the way group processes are interactionally brought into being and to get an analytical grip on them. In combination with Positioning Theory and treating the target of the displayed stances as the positioning object, it is not only possible to trace how these practices are situated in the sequential unfolding of the interaction, but also to describe implicit references to topic-related discourses. Hence, by systematically relating micro- and macro-analytical findings to each other dynamic communalization and distinction activities can be described and interpreted. This procedure might be transferred to other pre-determined topics discussed by focus groups. The framework been proven especially fruitful for studying focus group interactions where all members usually participate in conversation about one topic. However, the framework might also serve as a useful description tool in further research projects for studying dynamics in more naturalistic group interaction settings.

## Data Availability Statement

The datasets are not completely available because the participants did not give written consent to share the videos. However, all of the transcripts of the data analyzed for this study (in German language) can be found on the following website: https://www.degruyter.com/document/isbn/9783110727845/html, licensed as CC BY NC ND.

## Ethics Statement

Ethical review and approval was not required for the study on human participants in accordance with the local legislation and institutional requirements. Written informed consent to participate in this study was provided by the participants’ legal guardian/next of kin.

## Author Contributions

KW-Z agrees to be accountable for the content of the work.

## Conflict of Interest

The author declares that the research was conducted in the absence of any commercial or financial relationships that could be construed as a potential conflict of interest. The reviewer JS declared a past co-authorship/collaboration with the author KW-Z to the handling editor.

## Publisher’s Note

All claims expressed in this article are solely those of the authors and do not necessarily represent those of their affiliated organizations, or those of the publisher, the editors and the reviewers. Any product that may be evaluated in this article, or claim that may be made by its manufacturer, is not guaranteed or endorsed by the publisher.

## Footnotes

1 A programmatic view on turns, questioning: “Why that now?” and “What comes next?”

2 A second order positioning is established when the first positioning is rejected, while a third order positioning refers to the subsequent thematization of a positioning.

3 Stance lead refers to the first individuals’ positioning, offering orientation to the other individual. Stance follow refers to the second individual, who positions themselves, aligning with the first speaker.

4 The pattern *Catalogue* refers to a sequence of turns of participants who respond to a request of the moderator, providing their answers one by one, without necessarily reacting to the content of the previous partners’ talk. The pattern Gavora calls the *Domino* encompasses the participants responding to each other.

5 See the dispute between Schegloff (1997, 1999); Wetherell, 1998, and Billig, 1999; as summarized by Korobov (2001).

6 Although Du Bois draws on the stance concept as suggested by Ochs (1996) who acknowledges both, epistemic and affective stance taking as essential.

7 Shae is the name of a character played by Sibel Kekilli.

8 Nor does he in the following sequences about *Game of Thrones* in the course of the group discussion.

9 He continues referring to episodes, seasons and series titles all as “broadcast” throughout the discussion.

10 However, from a critical discourse analytic perspective, this is also meant to be seen critical, since series labeled as “quality TV” typically refer to US-American high budget productions for an educated audience (see Staiger, 2019, p. 174).
